# Role of Proton Pump Inhibitor on Esophageal Carcinogenesis and Pancreatic Acinar Cell Metaplasia Development: An Experimental *In Vivo* Study

**DOI:** 10.1371/journal.pone.0112862

**Published:** 2014-11-21

**Authors:** Luigi Dall’Olmo, Matteo Fassan, Elisa Dassie, Marco Scarpa, Stefano Realdon, Francesco Cavallin, Matteo Cagol, Giorgio Battaglia, Marco Pizzi, Vincenza Guzzardo, Erica Franceschinis, Gianfranco Pasut, Massimo Rugge, Giovanni Zaninotto, Nicola Realdon, Carlo Castoro

**Affiliations:** 1 Surgical Oncology Unit, Veneto Institute of Oncology (IOV-IRCCS), Padua, Italy; 2 ARC-NET Research Centre, University and Hospital Trust of Verona, Verona, Italy; 3 Department of Medicine, Surgical Pathology and Cytopathology Unit, University of Padua, Padua, Italy; 4 Endoscopy Unit, Veneto Institute of Oncology (IOV-IRCCS), Padua, Italy; 5 Venetian Institute of Molecular Medicine (VIMM), Padua, Italy; 6 Pharmaceutical Chemistry and Pharmacology Department, University of Padua, Padua, Italy; 7 Department of Medicine (DIMED), University of Padua, Padua, Italy; 8 Department of Surgery & Cancer, Imperial College of London, London, United Kingdom; Shiga University of Medical science, Japan

## Abstract

Chronic gastro-duodenal reflux in the esophagus is a major risk for intestinal metaplasia and Barrett’s adenocarcinoma. A role for chronic use of proton pump inhibitor (PPI) in the increased incidence of esophageal adenocarcinoma in Western countries has been previously suggested. The aim of this work was to study the effect of chronic administration of omeprazole (a proton pump inhibitor) *per os* in a model of reflux induced esophageal carcinogenesis. One week after esophago-gastro-jejunostomy, 115 Sprague-Dawley rats were randomized to receive 10 mg/Kg per day of omeprazole or placebo, 5 days per week. The esophago-gastric specimens were collected 28±2 weeks after randomization and analyzed in a blinded fashion. Mortality and esophageal metaplasia rates did not differ between the two groups (p = 0.99 for mortality, p = 0.36 for intestinal metaplasia and p = 0.66 for *multi-layered epithelium*). Gastric pancreatic acinar cell metaplasia (PACM) was more frequently observed in PPI-treated rats (p = 0.003). Severe ulcer lesions significantly prevailed in the placebo group (p = 0.03). Locally invasive esophageal epithelial neoplasia were observed in 23/39 PPI-treated versus 14/42 placebo-animals (p = 0.03). In conclusion, chronic omeprazole treatment improved the healing of esophageal ulcerative lesions. Locally invasive neoplastic lesions and PACM prevailed among PPI-treated animals. However, neither an effect on the overall mortality nor on the incidence of pre-neoplastic lesions was observed in this work.

## Introduction

Barrett’s carcinogenesis is a multi-step process from esophageal normal squamous mucosa to adenocarcinoma, through metaplastic intestinalized epithelium (i.e. Barrett’s epithelium) and dysplastic stages [1], [2].

Epidemiology of esophageal cancers has been changing over the last 30 years, since the introduction and wide diffusion of gastric acid suppressors among patients with gastro-esophageal reflux disease (GERD) in Western Europe and USA. A rapid increase of esophageal adenocarcinoma (EAC) and gradual decrease of esophageal squamous cell carcinoma (ESCC) has been extensively reported in this geographic area, particularly among white, male adults [3],[4].

GERD is generally accepted as a major risk factor for EAC and since acid suppressors can modify the composition of the refluxate, mainly its pH, it has been proposed that the use of those drugs could be responsible for the dramatic increase in the incidence of EAC [5].

Proton pump inhibitors (PPI) are a class of very efficient acid suppressors. They are usually able to control GERD symptoms and prevent its complications, mainly esophageal inflammation and strictures. However, concerns that PPI-induced hypergastrinaemia may increase the risk of adenocarcinoma development have also been expressed [6]. *In vitro* studies have shown that gastrin has proliferative effects on Barrett’s epithelium [7]. A potential causal effect of gastrin on neoplastic progression in human Barrett’s esophagus (BE) has been supported by a study showing that serum gastrin levels were significantly correlated with cellular proliferation in nondysplastic BE patients on PPI therapy [8].

On the contrary, a preventive role of PPI in Barrett’s adenocarcinogenesis has also been proposed, based on laboratory data of both *in vitro* and *ex vivo* experiments. However, *in vivo* models of reflux carcinogenesis have not revealed a reduction in adenocarcinoma risk in animals treated with proton pump inhibitors [9], [10]. Therefore, the effect of acid suppressors on Barrett’s esophagus and esophageal adenocarcinoma is still under debate [11].

The aim of this study was to investigate the role of omeprazole, a proton pump inhibitor, in a reflux rat model of esophageal carcinogenesis.

## Materials and Methods

### Animal groups

All procedures were conducted according to Italian law on the use of experimental animals (DL n. 116/92 art. 5). This study was approved by the Ethical Committee of Padua University (Comitato Etico di Ateneo sulla Sperimentazione Animale-CEASA). In this study, 115 male Sprague Dawley rats (Charles River, Lecco, Italy) were consecutively submitted to a surgical procedure to induce gastro-esophageal reflux (GER). The animals were kept under standard laboratory conditions (room temperature 22±2°C, 55±5% humidity, 12 h light-dark cycle) and acclimatized for at least a week before the procedure.

Water and standard chow were given *ad libitum* before surgery. Water was permitted 2 hours after surgery and rat chow was provided on the following day.

Postoperatively, the animals were housed one to a conventional cage. After the operation, they were randomly divided into two study groups PPI (n = 57) and Placebo (n = 58) using a computer-generated sequence. PPI group received chow containing 10 mg/Kg per day of omeprazole (Antra, ASTRAZENECA SpA) 5 days per week. The dose of 10 mg/kg per day of omeprazole was based on previous experimental literature data [12], [14] on the same species. Moreover, in a previous pilot study by our group (unpublished data), omeprazole dose of 10 mg/Kg daily was effective in increasing intra-gastric pH from 2–3 to 4–5 in non-operated rats, which is comparable to the therapeutic effect in humans. In fact, we found that rats are extraordinarily resistant to proton pump inhibitors with respect to the inhibition of gastric acid secretion, as already described for mice [15].

### Postoperative animal care

In the first month after surgery, the animals were monitored daily, then at least weekly, to follow up their clinical conditions and ascertain their therapeutic needs.

In the first week after surgery, we adopted a drug administration protocol consisting of an infusion of analgesic (Contramal® 5 mg/kg t.i.d.), antibiotic (Depotyl-LA® 20 mg/kg every 3 days) and fluids (saline solution 5 ml t.i.d. and Stimovit® 1,5 ml b.i.d., subcutaneously). After the 1st week after surgery, drug administration (analgesics and fluids) was based on each animal’s welfare score and general condition. Animals showing altered clinical condition were checked more frequently and treated with analgesics or fluids.

In this study, a numerical welfare scoring system (NWS) was used to assess pain, distress and discomfort after surgery. The NWS assigned a value (from 0 = normal to 3 = severely abnormal) to five different parameters, i.e., body weight loss more than 20%, appearance, clinical signs, and spontaneous and provoked behavior, as previously described in other studies [16]. The NWS score resulted from the sum of the five values obtained at the clinical visit.

A protocol of premature euthanasia for humane reasons was established for all animals, either scoring a NWS>6 at 2 weeks after surgery or showing a body weight loss exceeding 30% of the preoperative weight, throughout the experiment.

An independent veterinary assessment established any need for further premature euthanasia whenever the animal’s clinical condition suggested severe suffering.

### Anesthesia and surgical procedure

As previously reported [17], anesthesia was given using isofluorane (Forane®, Abbott S.p.A., Campoverde, MI, Italy) 3% for induction and 1.5% for maintenance, and oxygen 1 l/min. The animals were given 5 mg/kg of Tramadol (Contramal®, Formenti, Verona, Italy) intraperitoneally immediately after the peritoneal incision. At the end of the surgical procedure, the animal was roused, maintaining 1 l/min oxygen. The animals received 5 ml saline solution subcutaneously and intramuscular injections of tylosin 20 mg/kg (Depotyl-LA®) to prevent dehydration and surgical infections. None of the above-mentioned drugs are known as carcinogens.

The operation was performed according to the microsurgical procedure previously described by our group [18]. Briefly, a 1.5 cm side-to-side surgical esofago-gastric-jejunal anastomosis was created between the first jejunal loop and the gastro-esophageal junction, about 3 cm distal to Treitz’s ligament, with accurate mucosa-to-mucosa opposition, so that jejunal and gastric contents flowed back into the esophagus.

The surviving animals were killed at 28±2 weeks after surgery.

### Pathology

Immediately after death, the thoracic and abdominal cavities were examined and the esophagus, stomach, and jejunum were excised *en bloc*. The esophagus was opened longitudinally through the dorsal wall. With the mucosal surface uppermost, the margins of the specimen were fixed to a polystyrene plate with pins. Gross specimens were fixed in 10% neutral-buffered formalin for 24 hours. All specimens were examined grossly and cut serially (2–3 mm thick coronal sections). The tissue samples were routinely processed. Tissue sections (4 µm thick) were obtained from paraffin blocks and stained with haematoxylin & eosin (H&E). Lung and liver tissues were also grossly examine for metastases. Two experienced gastrointestinal pathologists (MR & MF) reviewed the slides in a blinded fashion.

Histology lesions were grouped into seven main categories (Table 1, Figure 2) [19]–[21]:

**Table 1:** Incidence of pathological findings observed in the animals under PPI treatment and in animals under placebo that reached the end of the experiments.

	PPI Group	Placebo Group	p-value^#^
N	39	42	-
Severe ulcerative lesions	7 (18)	17 (40)	0.03
Severe regenerative lesions	20 (51)	27 (64)	0.27
Intestinal Metaplasia (BE)	38 (97)	38 (90)	0.36
Multi-Layered Epithelium	17 (44)	21 (50)	0.66
Pancreatic Acinar Cell Metaplasia	22 (56)	10 (24)	0.003
Epithelial Neoplasia	23 (59)	14 (33)	0.03

Data expressed as n(%). ^#^Fisher Test. A p-value<0.05 is considered statistically significant. BE = Barrett esophagus.

**Figure 1:**
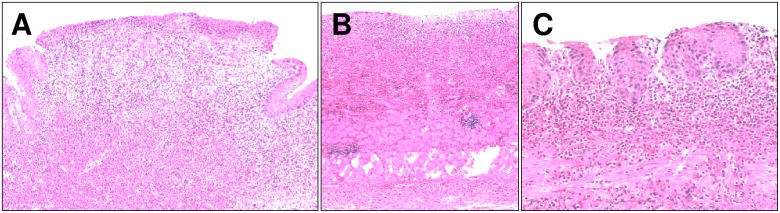
Representative images of esophageal ulcerative lesions (H&E stain). (A) severe ulcer; (B) severe and deep ulcer, up to the *muscolaris propria;* (C) superficial ulcer. Original magnification 20X (A and B) and 40X (C).

**Figure 2:**
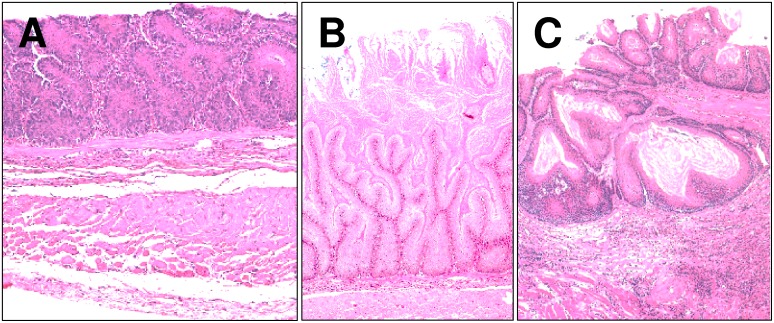
Representative images of esophageal lesions (H&E stain). (A) regenerative and (B and C) hyperplastic lesions within the esophageal mucosa. Original magnification 30X (A, B and C).

**Figure 3:**
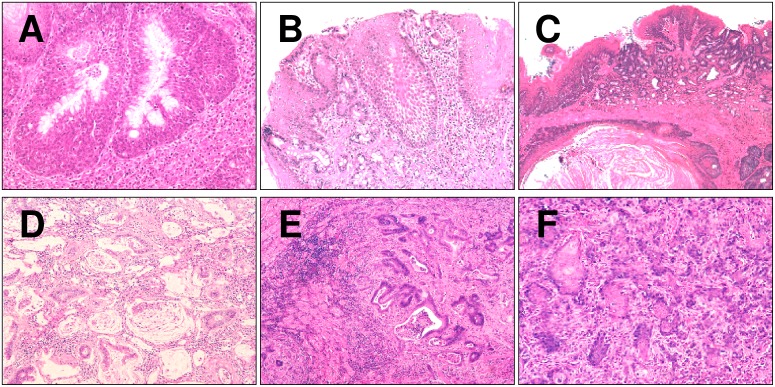
Barrett’s related lesions observed in the murine model (H&E stain). (A) multi-layered epithelium; (B) Barrett’s esophagus; (C) esophagistis *cystica profunda*; (D and E) esophageal glandular neoplasia; and (F) squamous cell neoplasia. Original magnification 40X (A), 30X (B), 20X (C–F).

**Figure 4:**
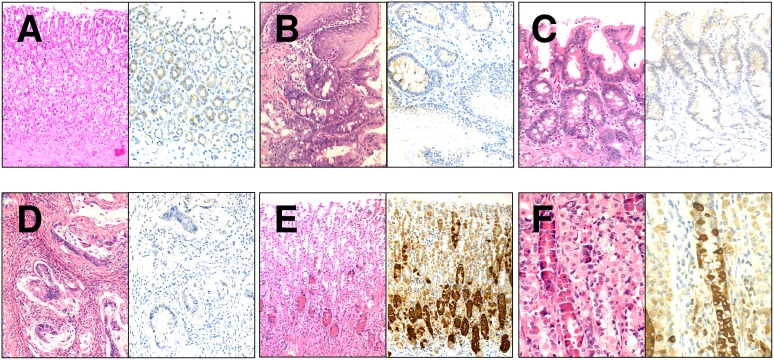
Representative images of α-amilase staining among gastroesophageal-mucosa samples. Examples of oxyntic mucosa (A), gastroesophageal mucosa with BE (B), BE (C), and EAC (D) showing a negative/weak immunoreaction. Pancreatic acinar cell metaplasia of the oxyntic mucosa was observed at higher incidence in the treated group and was strongly positive for α-amylase (E and F). Both H&E and the corresponding IHC staining are shown. Original magnification 20X, 40X.

1. Inflammatory lesions (further subdivided in non-ulcerative esophagitis and ulcer) (Figure 1). Non-ulcerative esophagitis was defined as sub-epithelial inflammatory infiltrate (mostly coexisting with intraepithelial leukocytes); micro-erosions were arbitrarily included in this category. Ulcers (defined as the complete loss of the mucosal layer with muscle exposure) were always coexistent with granulation tissue and hyperplastic-regenerative changes of the surrounding epithelium.
2. Regenerative-hyperplastic (also polypoid) lesions (Figure 2). Hyperplastic lesions were defined as thickening of the squamous epithelium (sometimes hyperkeratotic) with no cellular atypia. Regenerative lesions were assessed as increased length of the papillae, also coexisting with proliferative compartment hyperplasia (>20% of the mucosal thickness) [19]–[21].
3. Multi-layered epithelium (MLE) (Figure 3A). MLE consists of four to seven layers of cells that appear as basaloid squamous cells in the basal part and as columnar cells in the superficial layer. Therefore, MLE is a hybrid epithelium in which both squamous and columnar epithelia coexist and is considered a “proto-metaplasia” (i.e. a precursor of BE). Consistently with its phenotype, MLE expresses markers of both squamous and columnar differentiation [22]. The presence of MLE has been associated with reflux [5].
4. Intestinal metaplasia of the native squamous esophageal epithelium (i.e. Barrett’s esophagus, Figure 3B), defined by the presence of goblet epithelial cells [5], [19]–[21].
5. Esophagitis cystica profunda (Figure 3C), defined by the presence of jejunal cysts included within the esophageal wall.
6. Neoplasia. Neoplastic lesions featured a glandular pattern coexisting with mucous-lakes (mucinous cancer pattern, Figure 3D–E), or solid nests of epithelia with focal glandular differentiation or consisting of squamous well epithelia (squamous cell neoplasia, Figure 3F). In some case, the neoplastic lesions featured both squamous and glandular differentiation (adeno-squamous neoplasia).
7. Pancreatic acinar cell metaplasia [23] (PACM) was mainly detected within oxyntic mucosa being defined as nests of glandular structures phenotypically resembling pancreatic acinic cells. The pancreatic call differentiation was further confirmed by immunostain for α-amylase (Figure 4).

### Immunohistochemistry (IHC)

Histology sections 3–5 µm thick were obtained from each paraffin block and stained using the automated Leica Microsystems Bond-Max (Leica, Wetzlar, Germany) for α-amylase (A36; Biomeda, USA), as previously described [23]. Normal rat pancreatic tissue was used as positive control and was run concurrently with the other specimens. The slides were then lightly counterstained with hematoxylin. A strong cytoplasmic reaction was considered positive.

### Statistical Analysis

Data were presented as rates and percentages. The comparisons among groups were performed using a Fisher test, with a significance level of p<0.05.

## Results

Fifty-seven animals were randomized to the Omeprazole (PPI) group, while 58 to the placebo group. During the experiment, 27 animals were humanely euthanized for welfare reasons (14 in the PPI and 13 in the placebo group) with a median endpoint of 2 weeks. These cases were excluded from the study. Seven cases were found dead in the cage (4 in the PPI group and 3 in the placebo group). By applying the humane endpoints described above (see M&M), we achieved a low mortality rate in both groups. Thirty-nine and 42 rats reached the end of the experiments in PPI and placebo groups, respectively. The survival rates did not differ significantly between the two groups (p = 0.99).

The incidence of pathological findings is summarized in Table 1. All animals of both groups showed ulcerative and regenerative lesions of different degrees. Among rats treated with omeprazole, the incidence of severe ulcerative lesions was statistically inferior than in the placebo groups (18% vs 40%, respectively; p = 0.03), while the significance for the severity of regenerative lesions was not reached, even if the trend was toward a beneficial effect for the omeprazole treated group in preventing regenerative lesions.

As previously described with use of omeprazole [23], cystic degeneration and acinar-like cells with red granules were detected at the base of the gastric mucosa. These cells were immunohistochemically positive for α-amylase, and the pattern of staining was similar to that of pancreas acinar cells (Figure 4).

No differences were obtained when pre-cancerous lesions (i.e. BE and MLE) were considered.

On the contrary, pancreatic acinar cell metaplasia and neoplastic lesions were more frequently found in the PPI group (p = 0.003 and 0.03, respectively).

The neoplastic lesions mainly consisted of well differentiated mucinous tumours (18 in the PPI group and 9 in the placebo group); in one case in the PPI-group, the neoplasia only consisted of misplaced (well differentiated) glandular structures. The other neoplastic lesions featured squamous differentiation, sometime coexisting with glandular foci (Adeno-squamous differentiation). Regional nodal metastases were never detected.

## Discussion

This study considered the effects of long-term PPI treatment in a rat model of gastro-esophageal reflux. When mortality or esophageal metaplasia rates were considered, no significant differences resulted when comparing PPI- group *versus* placebo-group.

On the contrary, we found a difference between the two study groups in terms of prevalence of ulcerative esophagitis, rates of pancreatic acinar cell metaplasia (PACM) and neoplasia. As expected, ulcers significantly prevailed in the placebo group, since PPI are recognised as very effective in ulcer healing. PACM is a metaplastic change of oxyntic mucosa that has been described to be associated with both reflux [5] and PPI treatment [23], consistently with the present results.

In the present study, esophageal neoplasia prevailed among the PPI-treated animals (the prevalent histotype was mucinous adenocarcinoma). In a similar reflux rat model published by Ten Kate and collaborators in 2006, the neoplastic nature of similar histology has been questioned and the Authors described it as “*mucinous tumors with cytologic characteristics of well-differentiated mucinous adenocarcinomas… without infiltrative growth*” [21]. In the present study, because of the concurrence of both cytology atypia and architectural disarrangement, these lesions have been considered as locally invasive neoplastic lesions (even in absence of regional metastasis).

Surgical anti-reflux treatments and acid-suppressors in humans aim primarily to relieve symptoms of GERD. Anti-reflux surgery, typically a Nissen fundoplication, may be offered to selected patients with proven reflux disease who are refractory to medical treatment or to those reluctant to take life-long medication. Surgery provides both effective symptom relief and healing of esophagitis and offers the advantage of reducing both acid and bile reflux, which may act synergistically in the pathogenesis of Barrett’s esophagus [24].

On the other hand, the main available drugs (H2 antagonists and PPI) reduce acid secretion, with a consequent strong stimulus for gastrin production by G cells. Gastrin acts via its receptor (CCK2R) primarily present on enterochromaffin-like cells and parietal cells, stimulating proton pump production in parietal cells. This justifies the recurrence of acid-related symptoms after the interruption of a chronic treatment with acid suppressors and leads the patients with GERD to be maintained on treatments for long periods or life-long. Additionally, patients on NSAIDs treatment for chronic pain are usually on prophylaxis with PPI or H2 antagonist, to prevent peptic ulcer complications.

Acid suppressors have been the most prescribed drugs worldwide since the introduction of cimetidine in the 1975 by Sir James W. Black, the Nobel laureate who invented H2 antagonists, working on affinity of substances for a key receptor in acid-peptic disease (H2 receptors on parietal cells in the stomach). This fact changed the *scenario* of peptic disease from a surgical to a pharmacological treatment perspective.

On the other hand, PPIs act on the final common pathway of gastric acid secretion, permanently inactivating the H+/K+ ATPase (proton pump) in the parietal cell.

Since their introduction in the late 1980s, PPIs have assumed the major role for the treatment of GERD and other peptic disorders. Nowadays, PPIs are among the most widely prescribed drugs in the world, due to their efficacy and safety [25].

Interest in the potential role of PPIs in the prevention of adenocarcinoma in Barrett’s esophagus has been based on experimental data showing that recurrent episodes of acid reflux may have harmful effects on esophageal cells. An *ex vivo* explant model have reported an increase in cell proliferation and related signaling pathways after pulsatile acid exposure [26].

Intermittent acidic exposure has also been reported to generate DNA double strand breaks in transformed and primary Barrett’s esophagus and adenocarcinoma cells [27]. In an *in vivo* study in humans, PPI treatment has been associated with increased cell differentiation and decreased proliferation, both considered major goals in cancer chemoprevention [28].

On the other hand, acid exposure has shown antiproliferative effects in non-neoplastic Barrett’s epithelial cells *in vitro*. These findings contradicted the results of prior *in vitro* and *ex vivo* studies. The authors suggested that the prescription of antisecretory drugs in dosages beyond those required to heal GERD symptoms and endoscopic signs could be detrimental [29].

The effect of proton pump inhibitors on Barrett’s esophagus and esophageal adenocarcinoma is as yet controversial and unclear, and animal models of reflux treated with proton pump inhibitor have not revealed a reduction in adenocarcinoma risk [9]–[11]. Wetscher and colleagues reported an increased risk of gastric adenocarcinoma induced by one year of omeprazole treatment in Sprague Dawley rats with duodeno-gastric reflux [30]. These results were confirmed in 2004 by Viste and collaborators, who showed an increased risk of gastric cancer development in rats with duodenogastric reflux, when treated long-term with lansoprazole [31].

In conclusion, the present study confirms the role of omeprazole in the healing of mucosal ulcers. We observed an increase in pancreatic acinar cell metaplasia of the oxyntic mucosa in the animals treated with PPI, as already described in humans. To date, a link between this metaplasia and gastric neoplasia development has never been demonstrated. This work does not suggest an effect of the drug on overall mortality and on the incidence of esophageal metaplasia development. Moreover, this study documenting an increased prevalence of esophageal carcinomas was obtained in animals receiving long-term PPIs. This result should be viewed cautiously due to both model and study limitations. Thus, further studies are needed to clarify the effect of acid suppression on esophageal carcinogenesis.

## References

[1] Shaheen NJ, Richter JE (2009) Barrett’s oesophagus. Lancet. Mar 7 373(9666): 850–61. 10.1016/S0140-6736(09)60487-6 19269522

[2] Rugge M, Pizzi M, Castoro C (2014) Definition of Barrett’s Esophagus Dysplasia: Are We Speaking the Same Language? World J Surg. Jul 12. [Epub ahead of print]. 10.1007/s00268-014-2692-y 25015727

[3] Hur C, Miller M, Kong CY, Dowling EC, Nattinger KJ, et al (2013) Trends in esophageal adenocarcinoma incidence and mortality. Cancer. 2013 Mar 15 119(6): 1149–58. 10.1002/cncr.27834 PMC3744155 23303625

[4] Rugge M, Zaninotto G, Parente P, Zanatta L, Cavallin F, et al. (2013) Barrett’s esophagus and adenocarcinoma risk: the experience of the North-Eastern Italian Registry (EBRA). Ann Surg. 2012 Nov; 256(5): 788–94; discussion 794–5. Erratum in: Ann Surg. Mar; 257(3): 576. D'amore, Emanuele [corrected to d’Amore, Emanuele S G]. 10.1097/SLA.0b013e3182737a7e 23095623

[5] PT Chandrasoma, TR DeMeester (2006) GERD: Reflux to Esophageal Adenocarcinoma. Academic Press, Burlington, Massachusetts 01803, USA.

[6] Wang JS, Varro A, Lightdale CJ, Lertkowit N, Slack KN, et al (2010) Elevated serum gastrin is associated with a history of advanced neoplasia in Barrett’s esophagus. Am J Gastroenterol. May 105(5): 1039–45. 10.1038/ajg.2009.629 PMC3139948 19904251

[7] Haigh CR, Attwood SE, Thompson DG, Jankowski JA, Kirton CM, e al (2003) Gastrin induces proliferation in Barrett’s metaplasia through activation of the CCK2 receptor. Gastroenterology. 2003 Mar 124(3): 615–25. 10.1053/gast.2003.50091 12612900

[8] Green DA, Mlynarczyk CM, Vaccaro BJ, Capiak KM, Quante M, et al (2011) Correlation between serum gastrin and cellular proliferation in Barrett’s esophagus. Therap Adv Gastroenterol. Mar 4(2): 89–94. 10.1177/1756283X10392444 PMC3105623 21694810

[9] Moore KH, Barry P, Burn J, Falk G (2001) Adenocarcinoma of the rat esophagus in the presence of a proton pump inhibitor: a pilot study. Dis Esophagus. 14(1): 17–22. 10.1111/j.1442-2050.2001.00145.x 11422300

[10] Hao J, Zhang B, Liu B, Lee M, Hao X, et al (2009) Effect of alpha-tocopherol, N-acetylcysteine and omeprazole on esophageal adenocarcinoma formation in a rat surgical model. Int J Cancer. Mar 15 124(6): 1270–5. 10.1002/ijc.24077 PMC2677378 19058177

[11] Miyashita T, Shah FA, Harmon JW, Marti GP, Matsui D, et al (2013) Do proton pump inhibitors protect against cancer progression in GERD? Surg Today. 2013 Aug 43(8): 831–7. 10.1007/s00595-012-0395-2 23111465

[12] Erickson RA, Bezabah S, Jonas G, Lifrak E, Tarnawski AS (1991) Chronic omeprazole treatment increases duodenal susceptibility to ethanol injury in rats. Dig Dis Sci. Jul 36(7): 897–904. 10.1007/BF01297138 2070702

[13] Miwa H, Oshima T, Sakurai J, Tomita T, Matsumoto T, et al (2009) Experimental oesophagitis in the rat is associated with decreased voluntary movement. Neurogastroenterol Motil. 2009 Mar 21(3): 296–303. 10.1111/j.1365-2982.2008.01221.x 19126182

[14] Yenişehirli A, Naseri E (2008) Omeprazole, lansoprazole and pantoprazole had no effect on blood pressure and electrocardiogram of anesthetized rat. Pharmacol Res. Jul 58(1): 65–71. 10.1016/j.phrs.2008.06.012 18647653

[15] Waldum HL, Brenna E, Martinsen TC (2000) Safety of proton pump inhibitors. Aliment Pharmacol Ther. Nov 14(11): 1537–8. 10.1046/j.1365-2036.2000.00859.x 11069327

[16] Lloyd M, Wolfensohn S (1998) Practical use of distress scoring systems in the application of humane endpoints. In: Hendriksen CFM and Morton DB (eds.) Proceedings of the International Conference on Humane Endpoints in Animal Experimentation for Biomedical Research, 22–25 November 1998, Zeist, The Netherlands, 48–53. Royal Society of Medicine Press Limited, London, 1998.

[17] Dedja A, Dall’Olmo L, Cadrobbi R, Baldan N, Fante F, et al (2005) Heterotopic cardiac xenotransplantation in rodents: report of a refined technique in a hamster-to-rat model. Microsurgery. 2005 25(3): 227–34. 10.1002/micr.20101 15744724

[18] Ingravallo G, Dall’Olmo L, Segat D, Fassan M, Mescoli C, et al (2009) CDX2 hox gene product in a rat model of esophageal cancer. J Exp Clin Cancer Res. Aug 7 28: 108. 10.1186/1756-9966-28-108 PMC3225830 19664209

[19] Su Y, Chen X, Klein M, Fang M, Wang S, et al (2004) Phenotype of columnar-lined esophagus in rats with esophagogastroduodenal anastomosis: similarity to human Barrett’s esophagus. Lab Invest. 2004 Jun 84(6): 753–65. 10.1038/labinvest.3700079 15094711

[20] Fein M, Peters JH, Chandrasoma P, Ireland AP, Oberg S, et al (1998) Duodenoesophageal reflux induces esophageal adenocarcinoma without exogenous carcinogen. J Gastrointest Surg. May-Jun 2(3): 260–8. 10.1016/s1091-255x(98)80021-8 9841983

[21] Buskens CJ, Hulscher JB, van Gulik TM, Ten Kate FJ, van Lanschot JJ (2006) Histopathologic evaluation of an animal model for Barrett’s esophagus and adenocarcinoma of the distal esophagus. J Surg Res. Oct 135(2): 337–44. 10.1016/j.jss.2006.04.023 16926029

[22] Glickman JN, Chen YY, Wang HH, Antonioli DA, Odze RD (2001) Phenotypic characteristics of a distinctive multilayered epithelium suggests that it is a precursor in the development of Barrett’s esophagus. Am J Surg Pathol. May 25(5): 569–78. 10.1097/00000478-200105000-00002 11342767

[23] Hagiwara T, Mukaisho K, Ling ZQ, Sugihara H, Hattori T (2007) Development of pancreatic acinar cell metaplasia after successful administration of omeprazole for 6 months in rats. Dig Dis Sci. May 52(5): 1219–24. 10.1007/s10620-006-9253-7 17357842

[24] Jolly AJ, Wild CP, Hardie LJ (2004) Acid and bile salts induce DNA damage in human oesophageal cell lines. Mutagenesis 19(4): 319–24. 15215332 10.1093/mutage/geh035

[25] Katzung BG, Masters S (2012) Basic and Clinical Pharmacology. 12th Edition. Lange Basic Science.

[26] Fitzgerald RC, Omary MB, Triadafilopoulos G (1996) Dynamic effects of acid on Barrett’s esophagus. An ex vivo proliferation and differentiation model. J Clin Invest. Nov 1 98(9): 2120–8. 10.1172/JCI119018 PMC507657 8903332

[27] Clemons NJ, McColl KE, Fitzgerald RC (2007) Nitric oxide and acid induce double-strand DNA breaks in Barrett’s esophagus carcinogenesis via distinct mechanisms. Gastroenterology. Oct 133(4): 1198–209. 10.1053/j.gastro.2007.06.061 17919494

[28] Ouatu-Lascar R, Fitzgerald RC, Triadafilopoulos G (1999) Differentiation and proliferation in Barrett’s esophagus and the effects of acid suppression. Gastroenterology. Aug 117(2): 327–35. 10.1053/gast.1999.0029900327 10419913

[29] Feagins LA, Zhang HY, Hormi-Carver K, Quinones MH, Thomas D, et al (2007) Acid has antiproliferative effects in nonneoplastic Barrett’s epithelial cells. Am J Gastroenterol. Jan 102(1): 10–20. 10.1111/j.1572-0241.2006.01005.x 17266684

[30] Wetscher GJ, Hinder RA, Smyrk T, Perdikis G, Adrian TE, et al (1999) Gastric acid blockade with omeprazole promotes gastric carcinogenesis induced by duodenogastric reflux. Dig Dis Sci. Jun 44(6): 1132–5. 10.1023/a:1026615905170 10389684

[31] Viste A, Øvrebø K, Maartmann-Moe H, Waldum H (2004) Lansoprazole promotes gastric carcinogenesis in rats with duodenogastric reflux. Gastric Cancer. 7(1): 31–5. 10.1007/s10120-003-0264-1 15052437

